# Prevalence and risk factors for psychotic symptoms in young, first-episode and drug-naïve patients with major depressive disorder

**DOI:** 10.1186/s12888-024-05517-5

**Published:** 2024-01-23

**Authors:** Yuxuan Wu, Xueli Zhao, Zhe Li, Ruchang Yang, Ruijie Peng, Yue Zhou, Xingzhi Xia, Hanxu Deng, Xiaobin Zhang, Xiangdong Du, Xiangyang Zhang

**Affiliations:** 1 Medical College of Soochow University, Suzhou, China; 2 Suzhou Guangji Hospital, The Affiliated Guangji Hospital of Soochow University, Suzhou, China; 3 Xuzhou Medical University, Xuzhou, China; 4 CAS Key Laboratory of Mental Health, Institute of Psychology, Chinese Academy of Sciences, 16 Lincui Road, Chaoying District, Beijing, 100101 China

**Keywords:** First-episode psychosis, Major depressive disorder, Psychotic symptoms, Young adults

## Abstract

**Background:**

Major depressive disorder (MDD) is a common psychiatric disorder worldwide. Psychotic depression has been reported to be frequently under-diagnosed due to poor recognition of psychotic features. Therefore, the purpose of this study was to reveal the rate and risk factors of psychotic symptoms in young, drug-naïve patients with major depressive disorder at the time of their first episode.

**Methods:**

A total of 917 patients were recruited and divided into psychotic and non-psychotic subgroups based on the Positive and Negative Syndrome Scale (PANSS) positive subscale score. Anxiety symptoms and depressive symptoms were measured by the Hamilton Anxiety Rating Scale (HAMA) and the 17-item Hamilton Depression Rating Scale (HAMD-17), respectively. Several biochemical indicators such as total cholesterol (TC), triglycerides (TG), high-density lipoprotein cholesterol (HDL-C), low-density lipoprotein cholesterol (LDL-C), fasting blood glucose (FBG), thyroid stimulating hormone (TSH), free triiodothyronine (FT3), and free thyroxine (FT4) were also measured.

**Results:**

The rate of psychotic symptoms among young adult MDD patients was 9.1%. There were significant differences in TSH (*p*<0.001), FBG (*p*<0.001), TC (*p*<0.0001), TG (*p* = 0.001), HDL-C (*p* = 0.049), LDL-C (*p* = 0.010), diastolic blood pressure (DP) (*p*<0.001), systolic blood pressure (SP) (*p*<0.001), and HAMD total score (*p*<0.001) between young MDD patients with and without psychotic depression. HAMD, TSH, TC, and severe anxiety were independently associated with psychotic symptoms in young adult MDD patients. In addition, among young MDD patients, the rate of suicide attempts in the psychotic subgroup was much higher than in the non-psychotic subgroup (45.8% vs. 16.9%).

**Conclusions:**

Our findings suggest that psychotic symptoms are common in young MDD patients. Several clinical variables and biochemical indicators are associated with the occurrence of psychotic symptoms in young MDD patients.

## Introduction

Major depressive disorder (MDD), also known as clinical depression or major depressive disorder, is a common psychiatric disorder found throughout the world. According to the World Health Organization (WHO), MDD was ranked as the third leading cause of disease burden in 2008 and is expected to rank first by 2030 [1]. Globally, the lifetime risk of MDD is approximately 11.1–14.6% [2]. Furthermore, the period most likely to have the first episode of MDD ranges from mid-adolescence to mid-40s, with an average age of onset of around 20 years (median 25 years (18–43)) [3]. MDD is typically characterized by loss of interest, anhedonia, fatigue, sleep disturbances, anxiety, and sexual and cognitive dysfunction [4, 5]. Many factors have been understood to contribute to the mechanisms of MDD, but no single model can comprehensively explain all aspects of the disorder [6]. Therefore, we focused on young adult depressed patients aged 18–35 years to explore their disease characteristics.

MDD with psychotic symptoms (hereafter referred to as psychotic depression) was initially considered a function of MDD severity rather than a separate disorder [7]. In the fifth edition of the Diagnostic and Statistical Manual of Mental Disorders (DSM-5), the diagnosis of psychotic depression is not related to severity [8]. A previous study showed that psychotic depression is frequently under-diagnosed in the emergency room and inpatient settings due to poor knowledge of psychiatric features [9]. More importantly, different definitions and assessment methods have led to different estimates of the frequency rate, which may lead to an underestimation of the true prevalence [10]. As early as 1981, researchers raised the question of whether psychotic depression should be considered as a diagnostically independent disease [11, 12]. This issue is still under discussion today [13, 14]. If psychotic depression is considered a separate disorder, then a combination of antidepressants and antipsychotics would be necessary, which has been reported to be more effective than treatment alone or placebo [15, 16]. In a sizable sample of adolescents and young adults with depression, a prevalence of psychotic symptoms of 27% was observed [17]. Previous studies have explored the differences between psychotic depression patients and non-psychotic depression patients in terms of demographic characteristics and clinical features. Some demographic differences, such as family history of psychosis [18–20], education [21, 22], gender [23, 24], and age [24, 25], have been observed, but not all studies have found the same results. For example, Wang et al. [20] found no difference between psychotic depression patients and non-psychotic depression patients in terms of age of first onset. In addition to differences in demographic and clinical characteristics, the different outcomes of psychotic depression patients and non-psychotic depression patients are also of interest to researchers. Psychotic symptoms are often considered to be indicators of poor prognosis. For example, it has been shown that depressive symptoms are more severe and relapse rates are higher in psychotic depression patients compared to non-psychotic depression patients [21, 26, 27].

In recent years, there has been an uptick in the number of cases of depression reported among people of early adulthood. According to the World Mental Health Report 2022, young adults aged 18–29 have the highest rates of depression [28]. As the number of young people suffering from depression continues to rise despite stagnant progress in diagnosis and mental health care, it is crucial that we address this rising problem. According to Lincoln’s study, depressed children and adolescents have an estimated 30% suicidal behavior, which is significantly higher than their healthy peers [29]. Suicidal behavior was observed with greater frequency and severity in young adult patients, resulting in poorer outcomes [30].

Therefore, investigating risk factors for psychotic depression patients in young people and providing appropriate interventions and treatments at an early stage are crucial to improving the prognosis of psychotic depression patients. In this study, we aimed to examine (1) the prevalence of psychotic symptoms in young adult MDD patients; and (2) the demographic and clinical characteristics of psychotic depression patients in young adults.

## Methods

### Subjects

This is a study for adult patients with MDD in the First Hospital of Shanxi Medical University in which we chose adolescent patients as the study population, and a total of 917 outpatients were recruited. Prior to enrollment, all subjects were given detailed information about the study and were asked to sign an informed consent form acknowledging that they had read and agreed with the study’s procedures. The protocol and informed consent for this study were approved by the Institutional Review Board, the First Hospital, Shanxi Medical University.

Based on a number of previous studies in China, Australia, and Sweden, we defined 18–35 years of age as young adults [31–35].

All patients met the following inclusion criteria: (1) 18 to 35 years of age; (2) meeting the Diagnostic and Statistical Manual of Mental Disorders, Fourth Edition (DSM-IV) diagnosis of MDD; (3) first episode patients with no prior medication; and (4) 17-item Hamilton Rating Scale for Depression (HAMD) score ≥ 24.

Exclusion criteria included: (1) met any other major Axis I disorder; (2) presence of organic brain disease, ongoing infection, and other serious physical illness; (3) presence of substance abuse or dependence (except for nicotine), as determined by self-reported substance use and medical records; and (4) pregnant and breastfeeding women.

### Clinical interview and measurement

Two psychiatrists were trained to assess the positive subscale of the Positive and Negative Syndrome Scale (PANSS), the HAMD, and the Hamilton Anxiety Rating Scale (HAMA). Inter-rater correlation coefficients for HAMD, HAMA, and PANSS scores all exceeded 0.8 after repeated measures. Sociodemographic data were collected using a detailed questionnaire including age, sex, marriage status, duration of illness, and body mass index (BMI).

Depressive symptoms were measured with the HAMD. The HAM-D cutoff point used to distinguish patients with MDD from those without MDD was 24 [36]. Anxiety levels were measured using the HAMA. Using a cutoff score of 29, subjects were classified as having or not having severe anxiety [37]. In addition, PANSS positive subscale score was used to measure psychotic symptoms. In this study, patients were defined as having psychotic symptoms when they scored 15 or higher on the PANSS positive subscale score [38]. Based on this criterion, all subjects were divided into a psychotic depression group and a non-psychotic depression group.

“Have you ever attempted suicide in your life?” This question was used to assess suicide attempts. If the answer was “yes”, additional details were collected: time of the suicide attempt, the method, and exact date of each suicide attempt.

### Physical and biochemical measurements

Participants fasted overnight before having blood drawn between 6:00 and 8:00 am. By 11 a.m. on the same day, all blood samples were transported to the hospital laboratory. Fasting biochemical parameters were measured, including total cholesterol (TC), triglycerides (TG), high-density lipoprotein cholesterol (HDL-C), low-density lipoprotein cholesterol (LDL-C), fasting blood glucose (FBG), thyroid stimulating hormone (TSH), free triiodothyronine (FT3), and free thyroxine (FT4).

### Statistical analysis

Normal distribution of the continuous variables was verified by the Kolmogorov-Smirnov one-sample test. Since all continuous variables conformed to the normal distribution, they were described using the mean ± standard deviation (Mean ± SD) after performing the Student’s t-test. Meanwhile, Chi-square test was used for categorical variables to distinguish the differences between psychotic and non-psychotic subgroups. Through Chi-square test and Student’s t-test, the variables that statistically differed between two groups were selected for further logistic regression. Gender was also taken as a covariate in the model. In addition, patients were categorized into three subgroups based on age: 18–21-year group, 22–27-year group, and 28–35-year group. Variables with statistically significant differences between the two groups were selected by the chi-square test and Student’s t-test, and these were then entered into the logistic regression. In order to investigate the risk factors for psychotic symptoms in young MDD patients, a binary logistic regression was applied on MDD patients with and without psychotic symptoms. The non-age-stratified model was controlled for gender and age, and the age-stratified models were controlled for gender. Multicollinearity between independent variables was determined by variance inflation factor (VIF), with VIF > 5 indicating multicollinearity. The area under the curve (AUC) of the receiver operating characteristic curve (ROC) was used to determine the discriminatory power of the developed model in distinguishing between young adult MDD patients with and without psychotic symptoms.

Data management and analysis were performed using SPSS (IBM SPSS 24.0, SPSS Inc.). Graphs were plotted using GraphPad Prism 8 (GraphPad Software, Inc.). *p*-values were set as two-tailed with a significance level of α = 0.05.

## Results

### Demographics of young MDD patients

Among the young MDD patients, 351(38.3%) were male and 566(61.7%) were female. Of the psychotic depression patients, 27(32.5%) were male and 56(67.5%) of them were female. The average age(Mean ± SD) in non-psychotic subgroup was 24.74 ± 5.43 years, and it was 24.13 ± 5.37 in psychotic subgroups. The mean value of BMI from two groups was 24.29 ± 2.02 and 24.44 ± 2.12, respectively. Most patients were senior high school students(47.9%) followed by college students(37.6%). Furthermore, no statistically significant difference was observed between the age, sex, BMI, and other basic demographic information for both groups of patients (all *p* > 0.05).

### Prevalence of psychotic symptoms and clinical variables in young MDD patients

The prevalence of psychotic symptoms in young MDD patients was 9.1% (83/917). As shown in Table 1, TSH (*p* < 0.001), GLU (*p* < 0.001), TC (*p* < 0.0001), TG (*p* = 0. 001), HDL-C (*p* = 0.049), LDL-C (*p* = 0.010), DP (*p* < 0.001), SP (*p* < 0.001), and HAMD (*p* < 0.001) were significantly different between psychotic and non-psychotic depression patients. In addition, the suicide attempt rate was much higher in the psychotic subgroup (45.8%) than in the non-psychotic subgroup (16.9%) (*p* < 0.001). Furthermore, psychotic depression patients were more likely to have severe anxiety than non-psychotic depression patients (all *P* < 0.001). However, FT3 and FT4 (both *P* > 0.05) were not statistically different between psychotic and non-psychotic depression patients.

**Table 1:** Demographic and clinical characteristics in young psychotic and non-psychotic depression patients

	Non-psychotic	Psychotic	t/x^2^	*p*
Patient, n (%)	834(90.9%)	83(9.1%)		
Age (years, Mean ± SD)	24.74 ± 5.43	24.13 ± 5.37	0.97	0.330
Hierarchical age, n (%)			1.375	0.503
18–21 (years)	309(37.1%)	36(37.6%)		
22–27	247(29.6%)	21(25.3%)		
28–35	278(33.3%)	26(31.3%)		
Sex, n (%)			1.276	0.259
Male	324(38.8%)	27(32.5%)		
Female	510(61.2%)	56(67.5%)		
HAMD (Mean ± SD)	29.80 ± 2.73	33.81 ± 2.68	-12.77	<0.001
TSH (uIU/mL, Mean ± SD)	4.63 ± 2.37	7.20 ± 3.01	-7.537^a^	<0.001
FT_3_ (pmol/L, Mean ± SD)	4.94 ± 0.74	4.88 ± 0.68	0.058	0.494
FT_4_ (pmol/L, Mean ± SD)	16.77 ± 3.09	16.86 ± 3.01	-0.095	0.789
FBG (mmol/L, Mean ± SD)	5.34 ± 0.63	5.61 ± 0.70	-3.59	<0.001
TC (mmol/L, Mean ± SD)	5.11 ± 1.10	5.69 ± 1.17	-4.61	<0.001
TG (mmol/L, Mean ± SD)	2.10 ± 0.98	2.48 ± 0.98	-3.40	0.001
HDL-C (mmol/L, Mean ± SD)	1.23 ± 0.28	1.17 ± 0.28	1.97	0.049
LDL-C (mmol/L, Mean ± SD)	2.90 ± 0.88	3.16 ± 0.85	-2.59	0.010
BMI (kg/m^2^)	24.29 ± 2.02	24.44 ± 2.12	-0.69	0.490
SP (mmHg, Mean ± SD)	113.88 ± 10.25	118.76 ± 9.07	-4.17	<0.001
DP (mmHg, Mean ± SD)	73.86 ± 6.01	76.82 ± 5.97	-4.28	<0.001
Severe anxiety, n (%)			279.03	<0.001
no	789(94.6%)	29(34.9%)		
yes	45(5.4%)	54(65.1%)		
Attempted suicide, n (%)			40.07	<0.001
no	693(83.1%)	45(54.2%)		
yes	141(16.9%)	38(45.8%)		
Education level, n (%)			0.371	0.946
Junior high school	51(6.1%)	6(7.2%)		
Senior high school	398(47.7%)	41(49.4%)		
College	315(37.8%)	30(36.1%)		
Postgraduate	70(8.4%)	6(7.2%)		

Note: FBG: Fasting blood glucose; FT3: free triiodothyronine; FT4: free thyroxine; TSH: thyroid-stimulating hormone; TC: total cholesterol; TG: triglyceride; HDL-C: high-density lipoprotein cholesterol; LDL-C: low-density lipoprotein cholesterol; HAMD: 17-item Hamilton Rating Scale for Depression; BMI: body mass index; SP: systolic blood pressure; DP: diastolic blood pressure

### Factors associated with psychotic symptoms in young MDD patients

**Fig. 1:**
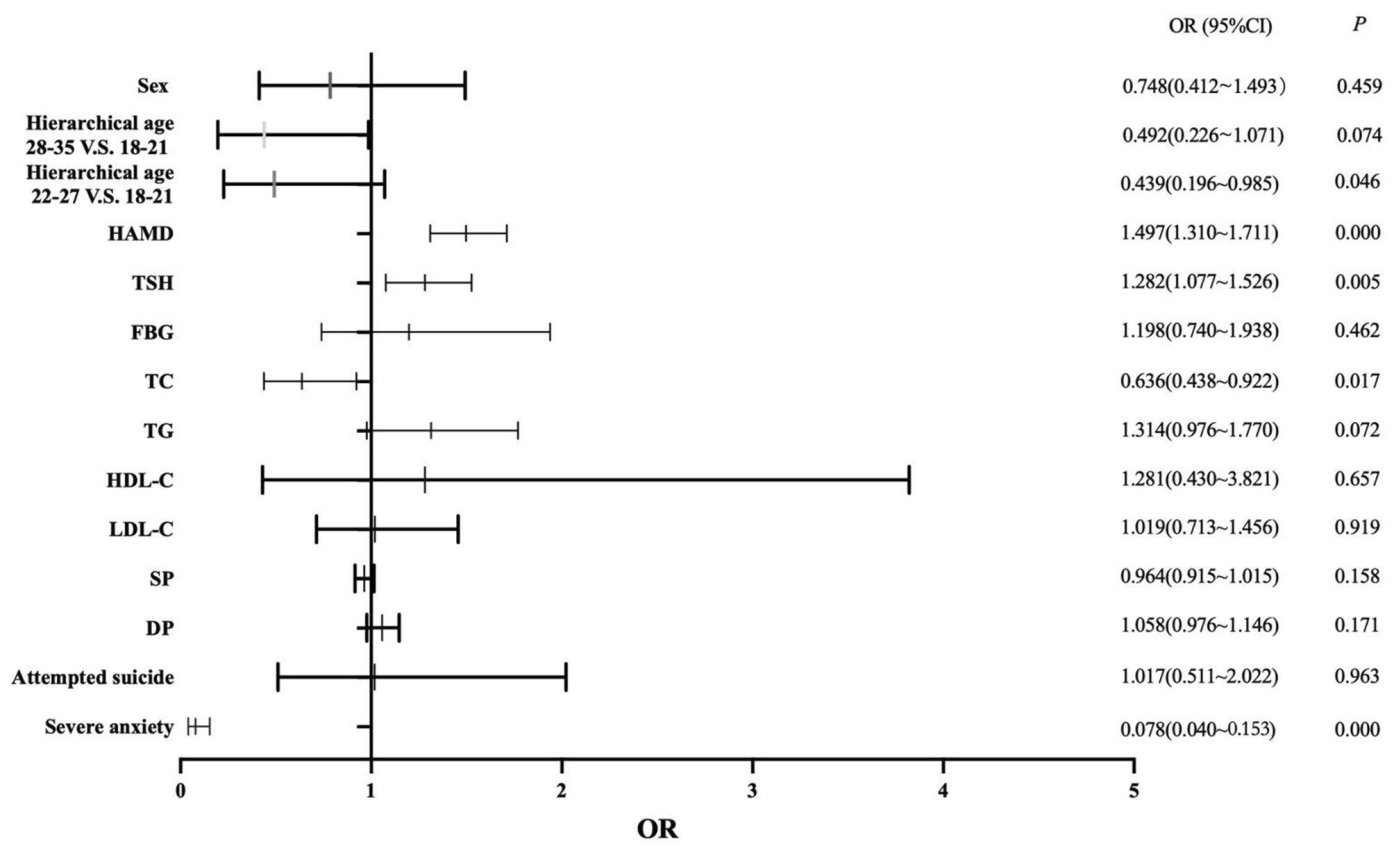
Factors associated with psychotic symptoms of young adult MDD patients. (Note: TSH: thyroid-stimulating hormone (uIU/mL); TC: total cholesterol (mmol/L); TG: triglyceride (mmol/L); HDL-C: high-density lipoprotein cholesterol (mmol/L); LDL-C: low-density lipoprotein cholesterol (mmol/L); HAMD: 17-item Hamilton Rating Scale for Depression; SP: systolic blood pressure (mmHg); DP: diastolic blood pressure (mmHg))

**Fig. 2:**
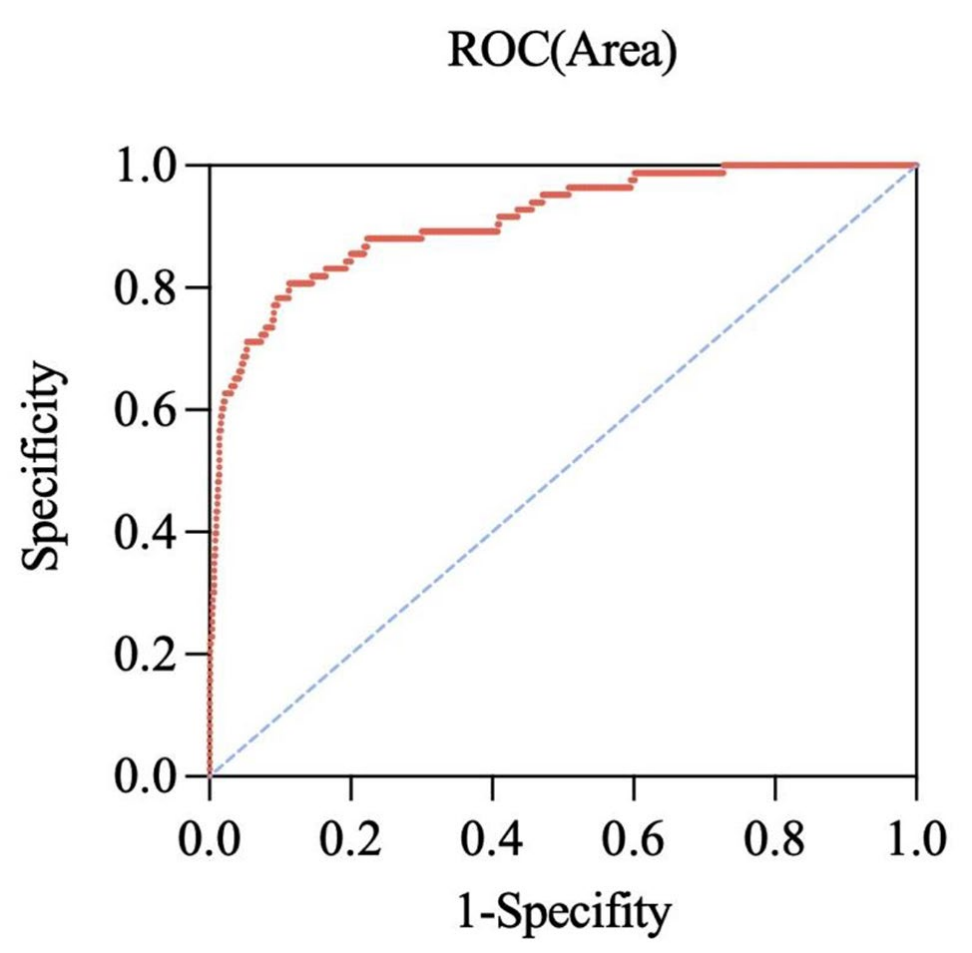
The ROC curves of risk factors for psychotic symptoms in young adult MDD patients. The area under the ROC curve was 0.905

**Fig. 3:**
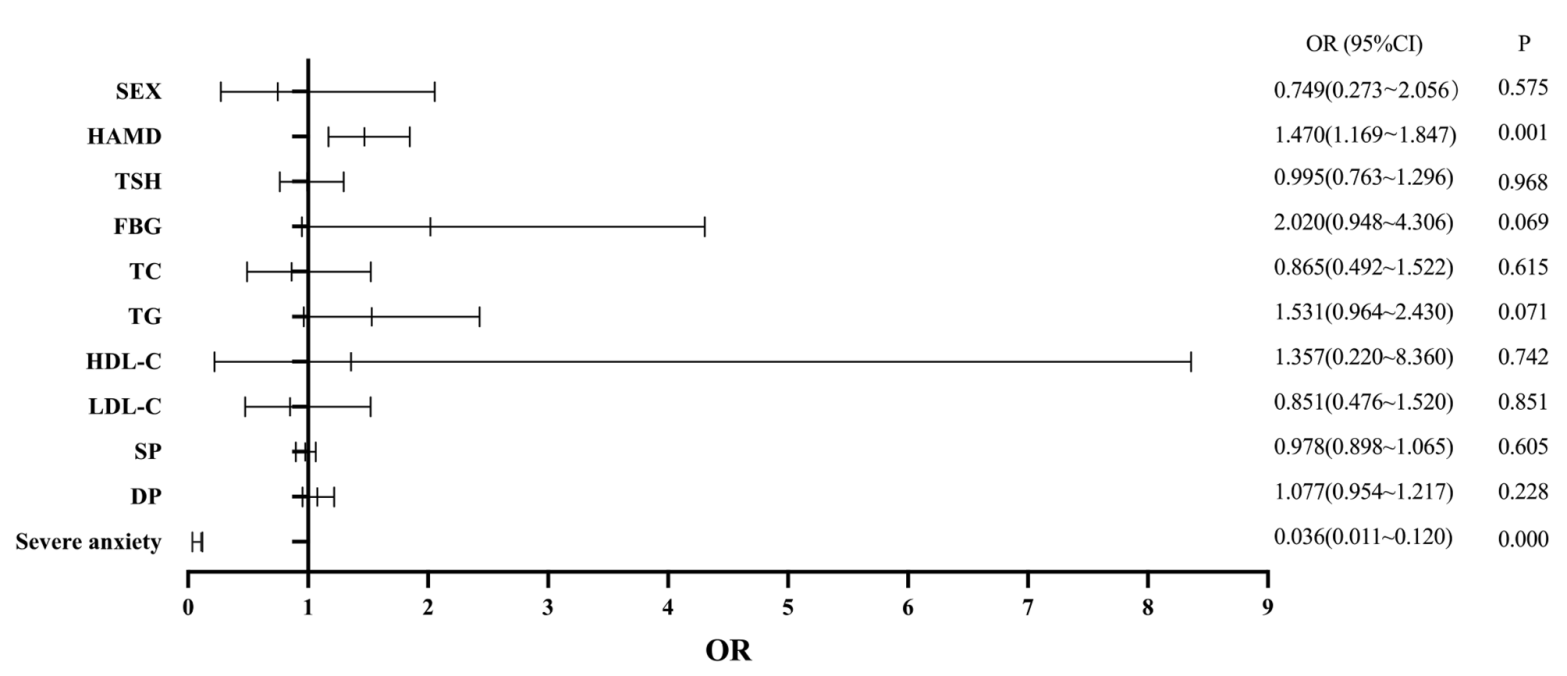
Factors associated with psychotic symptoms of young adult MDD patients aged 18–22 years. (Note: TSH: thyroid-stimulating hormone (uIU/mL); TC: total cholesterol (mmol/L); TG: triglyceride (mmol/L); HDL-C: high-density lipoprotein cholesterol (mmol/L); LDL-C: low-density lipoprotein cholesterol (mmol/L); HAMD: 17-item Hamilton Rating Scale for Depression; SP: systolic blood pressure (mmHg); DP: diastolic blood pressure (mmHg))

**Fig. 4:**
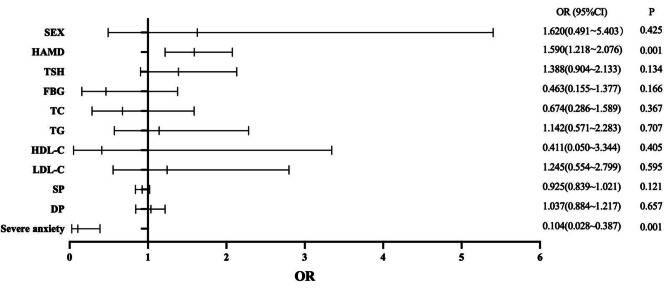
Factors associated with psychotic symptoms of young adult MDD patients aged 22–27 years. (Note: TSH: thyroid-stimulating hormone (uIU/mL); TC: total cholesterol (mmol/L); TG: triglyceride (mmol/L); HDL-C: high-density lipoprotein cholesterol (mmol/L); LDL-C: low-density lipoprotein cholesterol (mmol/L); HAMD: 17-item Hamilton Rating Scale for Depression; SP: systolic blood pressure (mmHg); DP: diastolic blood pressure (mmHg))

**Fig. 5:**
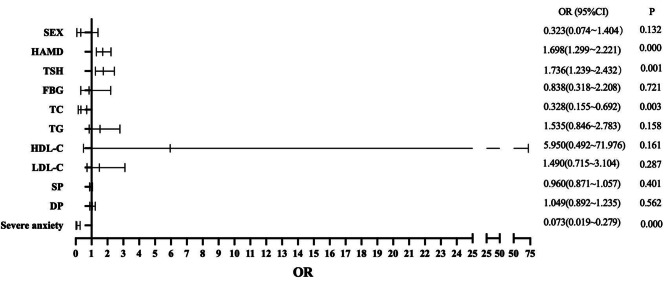
Factors associated with psychotic symptoms of young adult MDD patients aged 28–35 years. (Note: TSH: thyroid-stimulating hormone (uIU/mL); TC: total cholesterol (mmol/L); TG: triglyceride (mmol/L); HDL-C: high-density lipoprotein cholesterol (mmol/L); LDL-C: low-density lipoprotein cholesterol (mmol/L); HAMD: 17-item Hamilton Rating Scale for Depression; SP: systolic blood pressure (mmHg); DP: diastolic blood pressure (mmHg))

A binary logistic regression model was used to determine the factors associated with psychotic symptoms in young MDD patients. The results are exhibited in Fig. 1, showing that HAMD (OR = 1.497; 95% CI: 1.310–1.711), TSH (OR = 1.282; 95% CI: 1.077–1.526), TC (OR = 0.636; 95% CI: 0.438–0.922) and severe anxiety (OR = 0.078; 95% CI: 0.040–0.153) were independently associated with psychotic symptoms in young MDD patients. Furthermore, we found high AUC values (AUC = 0.910, *P* < 0.001; 95% CI = 0.874–0.946), which could distinguish psychotic depression patients from non-psychotic depression patients (Fig. 2). As shown in Figs. 3 and 4, no association was observed between TSH, TC, and psychotic symptoms at ages 18–21 or 22–27 when analyses were performed separately for the three different age categories. However, as shown in Fig. 5, the risk factors in the model for the 28–35-year group were consistent with the non-age-stratified model.

## Discussion

To our knowledge, this is the first study to investigate the prevalence of psychotic symptoms and associated factors among young FEDN MDD patients. This study revealed the following three main findings. (1) The prevalence of psychotic symptoms among young MDD patients was 9.1%. (2) The prevalence of suicide attempts was much higher in the psychotic subgroup than in the non-psychotic subgroup. (3) HAMD, TSH, TC, and severe anxiety were independently associated with psychotic symptoms in young MDD patients.

### The prevalence of psychotic symptoms among young MDD patients

We found that the prevalence of psychotic symptoms among young FEDN MDD patients was 9.1%. This result is consistent with the 9.2% prevalence of psychotic depression in Chinese MDD patients obtained from previous investigations [39]. In another previous study, a large sample size of Brazilian MDD patients showed a similar psychotic depression rate of 9.03% [40]. In addition, the prevalence of psychotic symptoms among French MDD patients was reported to be 11.2% [41], which is slightly higher than our finding. For adolescent patients, three previous studies reported psychotic depression rates of 18%, 27%, and 45%, respectively [17, 42, 43], higher than our results. Therefore, the prevalence of psychotic depression varies considerably among studies. These differences may be due to the following reasons, such as different ages (young adults, or adolescents, or adults) and countries of the study population, different methods of measuring psychotic symptoms, and different stages of the disease. For example, in Haley’s study, the DSM-III and the Diagnostic Interview Schedule for Children were used to define psychotic depression. Notably, in our study, psychotic depression was diagnosed using the PANSS positive subscale score. Therefore, it is reasonable to have different prevalence rates in each study when different criteria are applied. In addition, the young MDD patients in this study were included at the time of their first episode. It is possible that patients with MDD are more likely to develop psychotic symptoms over the long-term course of the disease, which may lead to a higher prevalence in patients with chronic MDD. In addition, it is possible that different ethnic and racial backgrounds contribute to differences in the prevalence of psychotic depression. As reported by Cohen et al., lifetime mortality rates are higher in Latinos and Blacks compared to Whites and Asians [44].

### The prevalence of suicide attempts in the psychotic subgroup and the non-psychotic subgroup among young adult patients

Our study showed that among young adult patients, the psychotic subgroup was more likely to have suicide attempts than the non-psychotic subgroup. This result is consistent with previous systematic reviews reporting that psychotic depression patients are twice as likely to attempt suicide as non-psychotic depression patients, both during their lifetime and during acute episodes [45, 46]. In addition, several other literatures also support our results [26, 47, 48] For example, Park et al. showed that psychotic depression patients were more likely to have a history of suicide attempts (41.7% vs. 22.1%) [26]. Also, Pawlak et al. observed an association between suicide attempts and psychotic symptoms in patients with MDD [48]. According to Fredriksen’s research, major depressive disorder patients may consider suicide as an escape from the psychotic symptoms [49]. A strong correlation between psychotic symptoms and perceived burdensomeness and thwarted belongingness may help explain why suicide attempts are more common in the psychotic subgroups [50]. Researchers found that those who had recently attempted suicide felt more burdened and less belonging than those who had not attempted suicide [51].

### Independent risk factors for psychotic symptoms in young MDD patients

We found that severe anxiety was independently associated with psychotic symptoms and was a significant protective factor for psychotic symptoms. Furthermore, among young depression adults, the psychotic subgroup was more likely to suffer from severe anxiety than the non-psychotic subgroup. These results are consistent with several studies that have shown a strong association between anxiety symptoms and psychotic symptoms in MDD patients [17, 26, 52]. For example, Park et al. revealed that psychotic depression patients had more severe anxiety [26]. Another study found that anxiety and depression were associated with a higher percentage of people exhibiting psychotic symptoms (approximately 27%) compared to 14% of the general population [17]. Therefore, we hypothesized that the presence of psychotic symptoms is a separate predictor of anxiety symptoms and a marker of the severity of young MDD patients.

Furthermore, higher TSH levels were observed to be independently associated with psychotic symptoms. Previous studies have shown that patients with psychotic depression have a considerably lower response to thyrotropin-releasing hormone stimulation compared with patients without psychotic symptoms [53]. Given the straightforward nature of this difference, we suggest that the higher TSH levels in the psychotic subgroup were caused by TSH insensitivity. Furthermore, psychotic depression patients scored much higher on HAMD than non-psychotic depression patients, which is consistent with many other studies. As demonstrated by Lattuada’s study [54–56], depressive symptoms were more severe in psychotic depression patients. Since this is the case, researchers may benefit from focusing on psychotic symptoms to assess the severity of MDD.

In contrast, TC levels were found to be a protective factor against psychotic symptoms in young MDD patients. Adiponectin is a hormone secreted almost exclusively by adipocytes [57]. A recent study found that adiponectin acted on 5-HT neurons via AdipoR1 receptors, thereby regulating depression-related behaviors in mice [58]. Lower levels of circulating adiponectin have been reported to be associated with more severe cases of depression [59]. Also, an inverse correlation was observed between serum adiponectin and TC [60]. Therefore, in the treatment of psychotic depression, early intervention by the therapist may be beneficial in young patients with more severe depression and higher TC levels in order to reduce psychotic symptoms and improve prognosis.

## Limitations

It is worth noting that our study has some limitations. First, it is difficult to infer causality from such a cross-sectional study. Longitudinal studies should be expanded to learn more about the variability and etiology of the disease. Second, the generalizability of our findings was hampered by the fact that our sample included only outpatients from one hospital in Shanxi. In our future study, we plan to recruit participants from both hospital and community settings. Third, we only used the PANSS positive subscale to classify patients into psychotic and non-psychotic depression, which may have excluded those with cognitive impairment, psychomotor disorders, and other psychosis-like symptoms. Thus, to diagnose psychotic depression more accurately in the future, researchers will need to use a battery of clinically and empirically validated diagnostic scales and factors to help identify patients with the aforementioned symptoms. In addition, suicidal ideation was not assessed by a validated scale in this study, and in our further research we will assess suicidal ideation using a specialized suicide rating scale, such as the Columbia Suicide Severity Rating Scale. Fourth, DSM-5 had not been used in China before this study was performed. Therefore, DSM-IV was used in this study, and we will use DSM-5 for further studies. Fifth, this study was conducted prior to the COVID-19 pandemic, which has had a significant impact on youth mental health. Further post-pandemic research is necessary to investigate the impact of the COVID-19 pandemic on youth mental health. In addition, a healthy control group will be essential in our future investigations.

## Conclusion

In conclusion, this study showed a prevalence of 9.1% of psychotic symptoms in young MDD patients. Furthermore, among young MDD patients, the rate of suicide attempts was higher in the psychotic depression patients than in the non-psychotic depression patients. In addition, a variety of independent risk variables were identified, including severe anxiety, HAMD, TSH, TC, and SP. As a result, high-risk groups require additional attention from psychiatrists. Clinical caregivers should closely monitor patients’ blood pressure. To keep track of psychotic symptoms, it is also important to monitor thyroid function and lipid metabolism, especially TSH and TC levels. The most important independent risk factor, severe anxiety, also warrants special attention. In this approach, the study offers new insights into early diagnosis and treatment options that may assist therapists identify young MDD patients who exhibit psychotic symptoms and modify treatment in a timely manner according to the course of the disease.

## Data Availability

The data are available from the corresponding author on reasonable request.
